# The In Vitro Analysis of Prebiotics to Be Used as a Component of a Synbiotic Preparation

**DOI:** 10.3390/nu12051272

**Published:** 2020-04-30

**Authors:** Katarzyna Śliżewska, Agnieszka Chlebicz-Wójcik

**Affiliations:** Institute of Fermentation Technology and Microbiology, Faculty of Biotechnology and Food Sciences, Lodz University of Technology, Wólczańska 171/173, 90-924 Łódź, Poland

**Keywords:** probiotics, prebiotics, inulin, synbiotics, microbial growth, metabolic activity, antagonism

## Abstract

Prebiotics are food components that are selectively fermented by beneficial microbiota and which confer a health benefit. The aim of the study was to select a prebiotic for the chosen probiotic strains to create a synbiotic. The impact of prebiotics (inulin, maltodextrin, corn starch, β-glucan, and apple pectin) on five *Lactobacillus* spp. strains’ growth and metabolites synthesis (lactic, acetic, propionic, and butyric acids, ethanol, and acetaldehyde) was tested by the plate count method and by high-performance liquid chromatography, respectively. Moreover, the differences in the ratio of D(−) and L(+) lactate isomers produced by *Lactobacillus* spp., as well as variations in the probiotics’ enzymatic profiles associated with the prebiotic used for cultivation, were determined with a Megazyme rapid assay kit and API^®^ ZYM assay, accordingly. Finally, the influence of the carbon source (prebiotic) used on the antagonistic activity of the probiotic strains towards pathogenic bacteria, such as *Salmonella* spp. or *Listeria monocytogenes* was analyzed in the co-cultures. The results showed that the growth, metabolic profile, and antagonistic activity of the probiotics towards selected pathogens were the most favorable when 2% (*w*/*v*) of inulin was used. Therefore, the combination of inulin with selected probiotics is a promising synbiotic mixture.

## 1. Introduction

Probiotics are defined as live strains of strictly selected microorganisms which, when administered in adequate amounts, confer a health benefit on the host [1]. On the other hand, in 1995, Glenn Gibson and Marcel Roberfroid introduced the concept of prebiotics, which were described as a “non-digestible food ingredient that beneficially affects the host by selectively stimulating the growth and/or activity of one or a limited number of bacteria in the colon, and thus improves host health” [2]. The International Scientific Association of Probiotics and Prebiotics (ISAPP) presented a new definition of a prebiotic in 2016, which characterized it as “a substrate that is selectively utilized by host microorganisms conferring a health benefit” [3].

Prebiotics are known for their positive effect on not only human but also animal health [4]. In order to define a carbohydrate as a prebiotic, in addition to being fermented by microorganisms, the compound should exhibit resistivity towards mammalian gastric enzymes and the acidity of the stomach [5]. On the other hand, prebiotics must be susceptible to the enzymes synthesized by bacteria residing in the colon [6]. What makes them different from other dietary fiber, such as xylans, and cellulose is that prebiotics favor the growth of only the beneficial microorganisms [7]. As a result of the prebiotics’ degradation, short-chain fatty acids (SCFAs) are produced, which is a desirable feature. SCFAs are organic acids which have fewer than six carbons in the chain, and the gastrointestinal tract (GIT) microbiota most commonly produce acetic, propionic, and butyric acids [8,9]. These short, volatile fatty acids can enter the bloodstream through enterocytes and can be metabolized in the brain or muscle tissue (acetate) or be utilized in the liver, interfering with cholesterol production or serving as the energy source for the GIT cells (butyric acid). Apart from this indirect mode of action, prebiotics can also modulate host health directly through GIT microbiota modulation [9,10,11]. Moreover, researchers have proven in both human and animal models that prebiotics could also hinder the attachment of pathogens to epithelial cells, have an impact on the secretion of the peptides responsible for appetite regulation, improve the absorption of minerals, and diminish the risk of colon cancer [12]. 

Besides probiotics and prebiotics being widely used, synbiotics are studied for their beneficial effect on host health. Synbiotics combine the pro- and prebiotic components in such a way that they act synergistically. In such preparations, prebiotics not only stimulate the growth of the beneficial microorganisms which already reside in the GIT, but they also enhance the survival of the included probiotics [13,14].

The following research aimed to compare the prebiotic potential of five substances—inulin, maltodextrin, corn starch, apple pectin, and β-glucan—in order to select the carbohydrate component for the creation of a synbiotic preparation with the previously selected probiotics. The impact of the carbohydrates on the growth of probiotics, SCFAs production, and antagonistic activity was assessed because these aspects are of vital importance to the prebiotic mechanism of action [15]. Moreover, the potentially negative effects of the selected carbohydrates were evaluated in comparison to glucose, by determining the concentrations of synthesized ethanol and acetaldehyde, and by characterizing the enzymatic profile. The described in vitro screening of the prebiotics poses as a valid basis for developing new synbiotic preparation, whose beneficial effect could be further studied in vivo.

## 2. Materials and Methods

### 2.1. Prebiotic Substances

Five different substances were analyzed for their prebiotic features: inulin from chicory roots (INU; Orafti^®^ HSI; BENEO–Orafti S.A., Oreye, Belgium), maltodextrin (MD; Sigma-Aldrich, St. Louis, MI, USA), apple pectin (AP; Sigma-Aldrich, St. Louis, Missouri, USA), 1,3/1,6 D β-glucan from brewer’s yeast (BG; Hepatica, Niemce, Poland) and corn starch (CS; RADIX-BIS Sp. z o.o., Rotmanka, Poland). For each analysis, glucose (GLU; Sigma-Aldrich, St. Louis, Missouri, USA) was used as the reference substance.

### 2.2. Microorganisms and Inoculum Preparation

The prebiotic effect was analyzed on 5 *Lactobacillus* spp. strains (Table 1), which were isolated with the use of Rogosa agar (BD Difco™, Sparks, NV, USA) from plant silage or the gastrointestinal tracts of monogastric animals within project no. PBS3/A8/32/2015. 

The strains are on deposit and can be purchased from the Łodź Collection of Pure Cultures (ŁOCK 105) of the Institute of Fermentation Technology and Microbiology, Łodź University of Technology [16]. The probiotic characteristics of selected strains of *Lactobacillus* spp. were previously described and are the subjects of patent applications [17,18,19,20,21].

In addition to that, four pathogenic strains were used: *Salmonella enterica* subsp. *enterica* serovar Typhimurium ATCC 13311, *Salmonella enterica* subsp. *enterica* serovar Enteritidis ATCC 13076, *Salmonella enterica* subsp. *enterica* serovar Choleraesuis PCM 2565, and *Listeria monocytogenes* ATCC 13932. The strains were obtained from the American Type Culture Collection (ATCC; Manassas, VA, USA) and the Polish Collection of Microorganisms (PCM; Wrosław, Poland).

All strains were stored in Cryobanks™ (Copan Diagnostics Inc., Murrieta, CA, USA) at −22 °C. Before analysis, both the probiotic and pathogenic strains were activated and passaged twice in de Man, Rogosa, and Sharpe broth (MRS; Merck Millipore, Darmstadt, Germany) or Nutrient broth (Merck Millipore), accordingly, at 37 °C for 24 h, without oxygen limitation. 

To prepare the inoculum, cells of each activated strain were washed twice in PBS (Calbiochem^®^, Merck Millipore) by centrifuging at 3468× *g* RCF (Centrifuge MPW-350R; MPW Med. Instruments, Warsaw, Poland) for 10 min, and afterward being re-suspended in PBS. A final optical density (OD) was set at a wavelength of 600 nm to values, which corresponded to the 1 × 10^8^ and 1 × 10^7^ colony-forming units per milliliter (CFU/mL) of probiotics and pathogens, respectively, based on previously prepared standard curves for each strain.

### 2.3. Growth of Selected Lactobacillus spp. Strains in the Presence of Prebiotics

Prebiotic impact on the *Lactobacillus* spp. strains’ growth was analyzed for five substances, namely, inulin, maltodextrin, corn starch, apple pectin, and β-glucan. The results were compared to the probiotic bacteria growth in the presence of glucose.

The prepared bacterial solutions of *Lactobacillus* spp. strains’ monocultures were added as 10% (*v*/*v*) inoculum to MRS broths, in which glucose was substituted with each prebiotic separately in concentrations of 1.0%, 1.5%, and 2.0% (*w*/*v*). MRS broths with 1.0%, 1.5%, and 2.0% (*w*/*v*) of glucose were used as the control medium. The cultures were incubated for 48 h at 37 °C. 

Subsequently, the serial dilutions were prepared, and the number of *Lactobacillus* spp. were determined with the use of the pour plate method in MRS agar after 48 h of incubation at 37 °C. Each culture was conducted in three repetitions and the results are presented as the CFU/mL.

### 2.4. Identification of Metabolites Produced by the Probiotic Lactobacillus spp. in the Process of Prebiotics Fermentation

The products of prebiotics fermentation by *Lactobacillus* spp. strains, namely, lactic acid, SCFAs (acetic, propionic, and butyric acids), acetaldehyde, and ethanol, were determined using high-performance liquid chromatography analysis (HPLC) on a liquid chromatograph Surveyor (Thermo Fisher Scientific Inc., Waltham, MA, USA).

Inoculums (10% *v*/*v*) of the probiotic strains’ monocultures were introduced into MRS medium with 1.0%, 1.5%, and 2.0% (*w*/*v*) of each prebiotic separately, as well as into MRS with an adequate concentration of glucose for the comparison. The strains were cultivated at 37 °C for 24 h without oxygen limitation. Subsequently, the samples were centrifuged for 10 min at 3468× *g* RCF, and 0.22 μm PTFE syringe filters (Millex-GS; Merck Millipore, Darmstadt, Germany) were used to filter them. Subsequently, 10 µL of each sample was injected into an Aminex HPX-87H column with a dimension of 300 × 7.8 mm (Bio-Rad Laboratories Inc., Des Plaines, IL, USA) by the loop dispensing valve with a syringe, which was used as an autosampler. The analysis was performed at 60 °C for 35 min per sample and 0.005 M of H_2_SO_4_ was used as a mobile phase (flow level: 0.6 µL/min).

The identification of the metabolites was performed in the chromatograms by comparing the retention times to the reference standards used to prepare standard curves. However, their concentrations were calculated based on the area underneath a specific peak. The analysis of each strain cultivated in the presence of different carbon sources was conducted three times.

### 2.5. Enzymatic Identification of Optically Active Forms of Lactic Acid

A D-/L-Lactic Acid (D-/L-Lactate) (Rapid) Assay Kit (Megazyme, Bray, Ireland) was used to assess the ratio of each of the optically active forms of lactic acid. The test was performed in accordance with the manual provided by the manufacturer.

Briefly, monocultures of selected strains’ inoculums (10% *v*/*v*) were cultivated in the MRS liquid medium in which glucose was replaced with 2% (*w*/*v*) of each of the prebiotic substances. Each strain’s monoculture grown in MRS liquid medium with 2% (*w*/*v*) glucose was used as the reference samples. The cultivation process was conducted at 37 °C for 24 h. Subsequently, the cultures were centrifuged for 10 min at 3468× *g* RCF, and the supernatants (50 µL) were submitted for analysis. Next, the 750 µL of sterile distilled water was added to each supernatant, as well as the solutions included in the test kit, such as a buffer (250 µL), NAD+ solution (50 µL), and D-GPT (10 µL). After 3 min of incubation, the optical density was measured (OD_1_) and the D-LDH (10 µL) was added. Samples were incubated for a further 5 min, and afterward, the OD_2_ was determined. Subsequently, 10 µL of L-LDH was added and the OD_3_ of the samples was measured after 10 min of incubation.

Each sample was analyzed in three repetitions and the fraction of D- and L-lactic acid was calculated based on the Equation (1):

c = 0.3204 × ∆OD_D_ + 0.3232 × ∆OD_L_ (1)

where “c” represents the total concentration of lactic acid, “0.3204 × ∆OD_D_” stands for the D-lactic acid concentration, and “0.3232×∆OD_L_” is the amount of the L(+) isomer, and where ΔOD_D_ = OD_2_ − OD_1_ and ΔOD_L_ = OD_3_ − OD_2_.

### 2.6. Enzymatic Profile of Probiotic Lactobacillus spp. in the Presence of Prebiotics

An API^®^ ZYM test kit (bioMérieux; Marcy l’Etoile, France) was used to establish differences in the enzymatic profile of probiotic *Lactobacillus* spp. strains depending on the carbon source used, such as prebiotics compared with glucose. The analysis was conducted according to the manual provided by the manufacturer of the test.

Monocultures of the selected strains were grown on MRS slants with each of the prebiotics separately or glucose comparatively at a concentration of 2% (*w*/*v*). The cultivation was conducted at 37 °C for 48 h. Afterward, the monocultures were suspended in sterile saline solution at a final density between 5 and 6 on McFarland’s scale. Each of the prepared bacterial suspensions was introduced individually into all microtubes of the API^®^ ZYM test strip and incubated at 37 °C for 4 h. Next, ZYM A and ZYM B reagents were added to every microtube and the test strips were exposed to light.

Every sample was analyzed in three repetitions and the positive results were obtained based on the color changes visible in the microtubes.

### 2.7. The Influence of the Prebiotics on the Antagonistic Activity of Lactobacillus spp. Strains towards Pathogenic Bacteria

The ability of the selected *Lactobacillus* spp. strains to inhibit the growth of pathogenic bacteria, namely *S*. Typhimurium, *S*. Enteritidis, *S*. Choleraeusis, and *L. monocytogenes*, was evaluated in co-cultures with the plate count method.

Monocultures of each probiotic or pathogenic strain were propagated in the MRS liquid medium or nutrient broth, respectively, with 2% (*w*/*v*) of the prebiotic substance, or glucose for comparison. The strains were cultivated at 37 °C for 24 h without oxygen limitation. Afterward, the bacterial monocultures grown on specific prebiotic were mixed in the amount of 1 mL of cultures of all probiotic strains with each pathogen separately. An initial number of bacteria was assessed by preparing serial decimal dilutions of each mixture, which were then used in the pour plate technique with MRS agar, Salmonella Shigella agar (SS agar; Merck Millipore), and PALCAM agar (Merck Millipore) for probiotics, *Salmonella* spp., and *L. monocytogenes*, respectively. Next, the co-cultures of probiotics and pathogens were incubated at 37 °C without oxygen limitation.

The changes in the bacterial count were evaluated after 4, 8, 12, and 24 h as it was previously done, in order to determine the initial number of bacteria. The plates were incubated at 37 °C for 24 h and the results are presented in CFU/mL. Each co-culture was conducted in three repetitions.

### 2.8. Statistical Analysis

Each of the statistical analysis was performed with XLSTAT Software (Addinsoft, SARL, Paris, France). The influence of the prebiotics on the growth performance of selected strains was analyzed with a multi-way analysis of variances (ANOVA) followed by post hoc Tukey’s test. Before the ANOVA, the data were checked for normal distribution with the Shapiro–Wilk test, and for the homogeneity of variances with Bartlett’s test. On the other hand, the correlation between the prebiotic used and the concentrations of metabolites produced by probiotic strains in the process of fermentation was checked with Principal Component Analysis (PCA) and is presented in the form of biplots including categorical variables and observations. Furthermore, the concentration differences of lactic acid isomers synthesized by the selected strains in the presence of chosen prebiotics were analyzed with the multi-way Welch-ANOVA test because the variances were not homogenous.

## 3. Results

### 3.1. Growth of Selected Lactobacillus spp. Strains in the Presence of Prebiotics

Selected *Lactobacillus* spp. strains showed similar growth in the presence of three different concentrations (1.0, 1.5, and 2.0% *w*/*v*) of 5 prebiotic substances, as well as when the glucose was used comparatively as the carbon source (Table 2). The abundance of probiotic strains varied between 7.53 ± 1.28 log CFU/mL and 11.09 ± 0.47 log CFU/mL. However, the numbers of *Lb. rhamno*sus ŁOCK 1087, *Lb. reuteri* ŁOCK 1092, and *Lb. plantarum* ŁOCK 0860 were significantly higher when inulin was used as the carbon source at a concentration of 2% (*w*/*v*): 10.71 ± 0.81, 11.09 ± 0.47, and 10.91 ± 0.92 log CFU/mL, respectively. A similar dependency was observed when 1.5% (*w*/*v*) of apple pectin was used for the cultivation of *Lb. pentosus* ŁOCK 1094 (10.84 ± 0.15 log CFU/mL).

### 3.2. Identification of Metabolites Produced by the Probiotic Lactobacillus spp. in the Process of Prebiotics Fermentation

**Figure 1:**
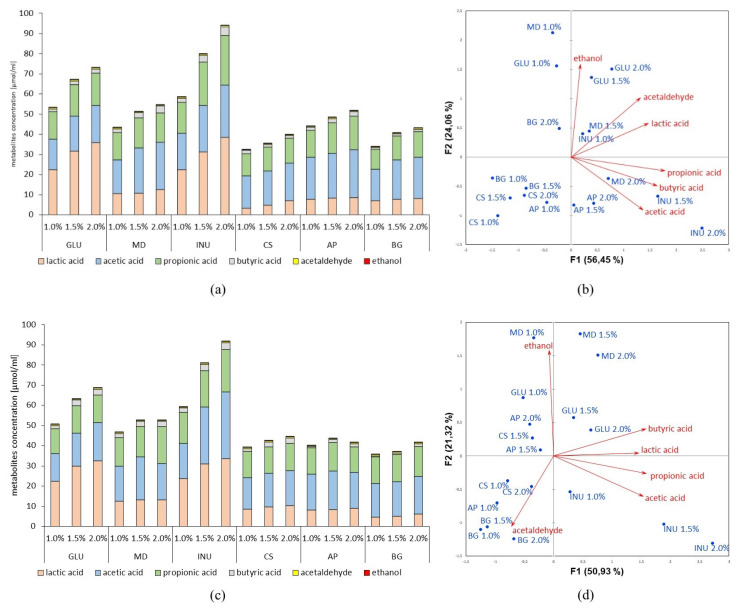

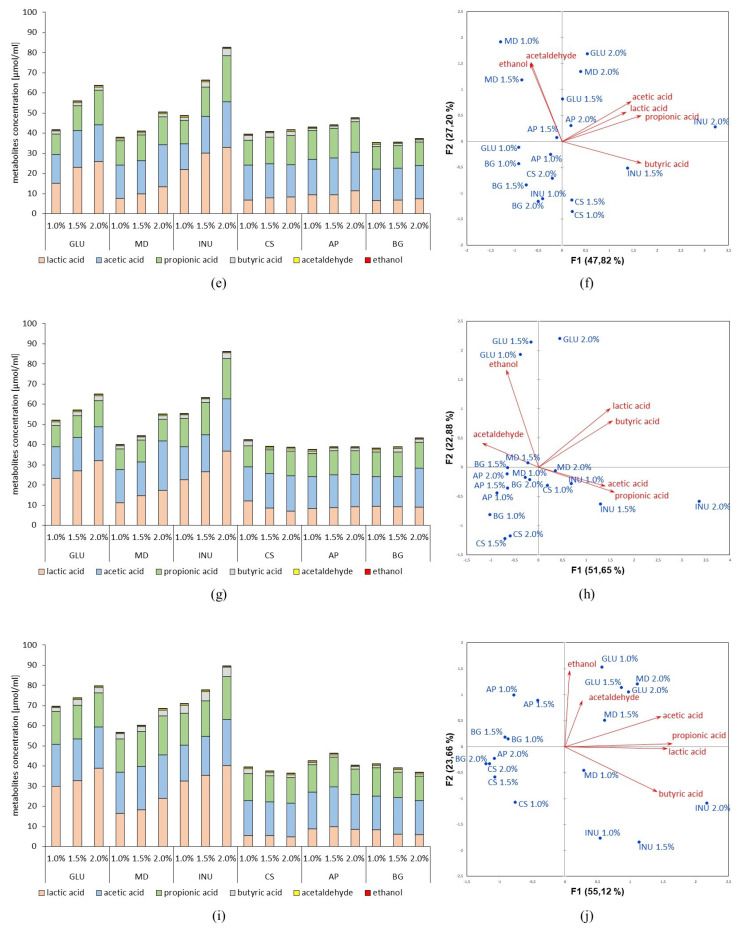
Metabolites concentration produced by probiotic strains, namely, (**a**) *Lb. rhamnosus* ŁOCK 1087, (**c**) *Lb. paracasei* ŁOCK 1091, (**e**) *Lb. reuteri* ŁOCK 1092, (**g**) *Lb. plantarum* ŁOCK 0860, and (**i**) *Lb. pentosus* ŁOCK 1094 in dependence on the used prebiotic (MD–maltodextrin, INU–inulin, CS–corn starch, AP–apple pectin, BG–β-glucan) or glucose (GLU) comparatively as a carbon source in the cultivation medium. Moreover, the correlations between used carbohydrates, as well as their concentrations, and the synthesized amount of metabolites, were presented as the PCA biplots, namely, plots (**b**,**d**,**f**,**h**,**j**) for above-mentioned strains, respectively.

Predominantly, the selected strains produced lactic, acetic, and propionic acids in the fermentation process of prebiotics and glucose. Nonetheless, butyric acid, acetaldehyde, and ethanol were also detected, although in substantially lower concentrations. Concentrations of the metabolites depended on the amount and type of carbon source used in the cultivation of probiotic monocultures (Figure 1).

The highest concentrations of lactic acid were observed when the strains were grown in the presence of 2% (*w*/*v*) inulin: the average amount was 30.71 µmol/mL (Figure 1a,c,e,g,i). Furthermore, increased secretion of SCFAs, namely acetic, propionic, and butyric acids, was observed; these compounds were produced in average concentrations of 21.29, 17.96, and 3.08 µmol/mL, respectively. These results indicate an enhancement of SCFAs production exceeding 20% due to usage of 2% (*w*/*v*) inulin as the carbon source in comparison to a similar concentration of glucose. Moreover, the data showed a high negative correlation between using inulin for the cultivation of the probiotic strains and ethanol, as well as acetaldehyde synthesis, which was the opposite for glucose (Figure 1b,d,f,h,j).

An unfavorable influence of the rest of the prebiotics studied, namely, maltodextrin, corn starch, apple pectin, and β-glucan, on the production of lactic acid and SCFAs by the probiotic strains was observed. A negative correlation between these prebiotics and lactic acid, as well as SCFAs, production was noted (Figure 1). 

It was observed that the most beneficial concentration of all prebiotics in terms of growth and metabolism was the 2% (*w*/*v*), which is why this concentration of carbohydrates was used in the further analysis.

### 3.3. Enzymatic Identification of Optically Active Forms of Lactic Acid

As a result of the analysis, both lactic acid isomers-D(−) and L(+)- were detected when the probiotic strains were grown in the presence of all tested prebiotics, and when glucose was used as the carbon source (Table 3).

The probiotic strains cultivated in the presence of corn starch, apple pectin, β-glucan, and maltodextrin synthesized significantly less total lactic acid than when glucose was used as the carbohydrate (Table 3). Moreover, no vital statistical differences in the concentrations of D(−) and L(+) isomers of this organic acid were observed when these prebiotics were used.

Significantly higher total concentrations of lactic acid produced by the analyzed strains were observed when inulin was fermented than in the presence of glucose. Furthermore, inulin had a beneficial impact on the ratio of optically active forms of lactic acid produced by the probiotic strains; the L(+) isomer was predominantly produced. The percentage share of L(+) form of lactic acid produced by most of the tested strains in the process of inulin degradation exceeded 93%, with the exception of *Lb. paracasei* ŁOCK 1091 (72.16%). An analogous dependency was noted when glucose was used as the carbon source for the strains, with the exception of *Lb. paracasei* ŁOCK 1091; however, the L-lactic acid percentages in the total amount of the metabolite were lower, varying between 35% and 86%.

### 3.4. Enzymatic Profile of Probiotic Lactobacillus spp. in the Presence of Prebiotics

Based on the API^®^ ZYM assay, it was observed that each strain exhibited activity of leucine arylamidase, acid phosphatase, naphthol-AS-BI-phosphohydrolase, β-galactosidase, as well as α- and β-glucosidase independent of the carbon source used for cultivation. However, the selected strains cultivated in the presence of glucose or prebiotics did not show lipase, trypsin, chymotrypsin, β-glucuronidase, α-mannosidase, or α-fucosidase activity (except for *Lb. rhamnosus* ŁOCK 1087). 

On the contrary, differences in the enzymatic profile of the selected probiotic strains in relation to the prebiotic substance used as the carbohydrate during cultivation were observed in terms of alkaline phosphatase and cystine arylamidase. The enzymatic profiles of *Lb. rhamnosus* ŁOCK 1087 and *Lb. pentosus* ŁOCK 1094 did not include alkaline phosphate when apple pectin was used as the carbon source. This enzyme activity was observed for *Lb. paracasei* ŁOCK 1091, *Lb. reuteri* ŁOCK 1092, and *Lb plantarum* ŁOCK 0860 when inulin, apple pectin, maltodextrin, or corn starch were used as carbohydrates in the cultivation process. However, cystine arylamidase activity was observed for *Lb. rhamnosus* ŁOCK 1087, *Lb. paracasei* ŁOCK 1091, *Lb. reuteri* ŁOCK1092, and *Lb. pentosus* ŁOCK 1094 when maltodextrin, inulin, or corn starch were used as the carbon source. Moreover, the activity of n-acetyl-β-glucosaminidase, which was carbon source-dependent was not observed only when inulin and glucose were fermented. 

The lowest enzymatic activity was observed for *Lb. plantarum* ŁOCK 0860, which was the only studied strain that did not present cystine arylamidase and α-galactosidase activity. Moreover, esterase, lipase esterase, and valine arylamidase activity was carbon source-dependent, where in the presence of apple pectin, β-glucan, and glucose, *Lb. plantarum* ŁOCK 0860 mostly did not exhibit those enzymes’ activity.

**Figure 2:**
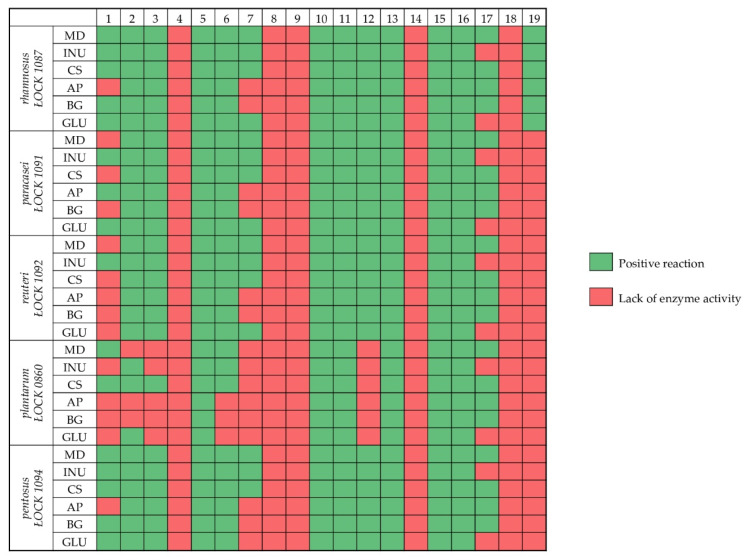
Heatmap presenting the *Lactobacillus* spp. strains enzymatic profile in relation to carbohydrates used in the culture: MD–maltodextrin, INU–inulin, CS–corn starch, AP–apple pectin, BG–β-glucan, and GLU–glucose. The tested enzymes were enumerated as follows: 1–alkaline phosphatase, 2-esterase (C4), 3-lipase esterase (C8), 4-lipase (C14), 5-leucine arylamidase, 6-valine arylamidase, 7-cystine arylamidase, 8-trypsin, 9-chymotrypsin, 10-acid phosphatase, 11-naphthol-AS-BI-phosphohydrolase, 12-α-galactosidase, 13-β-galactosidase, 14-β-glucuronidase, 15-α-glucosidase, 16-β-glucosidase, 17-n-acetyl-β-glucosaminidase, 18-α-mannosidase, 19-α-fucosidase.

The enzymatic activity results are presented in Figure 2.

### 3.5. The Influence of the Prebiotics on the Antagonistic Activity of Lactobacillus spp. Strains towards Pathogenic Bacteria

**Figure 3:**
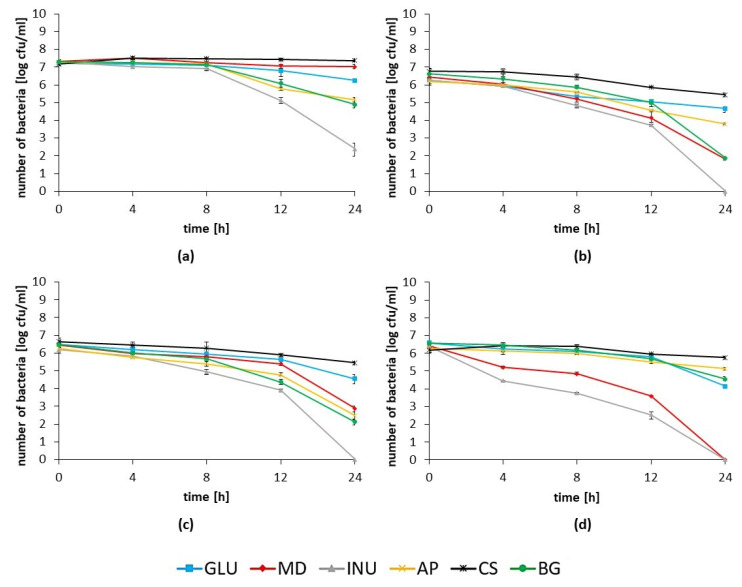
Antagonistic activity of a mixture of the probiotic strains, namely, *Lb. rhamnosus* ŁOCK 1087, *Lb. paracasei* ŁOCK 1091, *Lb. reuteri* ŁOCK 1092, *Lb. plantarum* ŁOCK 0860, and *Lb. pentosus* ŁOCK 1094 on the pathogenic bacteria: (**a**) *L. monocytogenes*, (**b**) *S.* Enteritidis, (**c**) *S*. Typhimurium, and (**d**) *S*. Choleraesuis. The co-cultures were conducted in the presence of each carbohydrate (MD–maltodextrtin, INU–inulin, AP–apple pectin, CS–corn starch, BG–β-glucan, or GLU–glucose).

The antimicrobial potential of a mixture of the *Lactobacillus* spp. strains, including *Lb. rhamnosus* ŁOCK 1087, *Lb. paracasei* ŁOCK 1091, *Lb. reuteri* ŁOCK 1092, *Lb. plantarum* ŁOCK 0860, and *Lb. pentosus* ŁOCK 1094 towards pathogens varied (Figure 3). Based on the results obtained, it was noted that the ability to reduce the number of pathogenic bacteria was dependent on the prebiotic used as a carbon source in co-cultures, as well as the type of pathogens.

The strongest antagonistic activity was observed when a co-culture with each pathogen was conducted in the presence of 2% (*w*/*v*) inulin, which resulted in a total reduction of the *Salmonella* spp. strains, being undetectable after 24 h of incubation (Figure 3b–d). The number of *L. monocytogenes*, however, was reduced from 7.29 logarithmic units to 2.39, which amounts to a reduction of almost 70% of the bacterial count (Figure 3a). 

On the other hand, corn starch used as the carbon source for the probiotic mixture culture resulted in a decline of these strains’ ability to reduce the number of all analyzed pathogenic bacteria in comparison to the effect observed when glucose was metabolized. An analogous dependency was observed in the probiotic mixture co-culture with *L. monocytogenes* in the presence of maltodextrin, as well as towards *S*. Choleraesuis when the apple pectin and β-glucan were used as the carbon sources (Figure 3a,d). 

Furthermore, the most susceptible pathogens to antagonistic activity were the analyzed *Salmonella* spp. strains, with the exception of the co-cultures cultivated in the presence of apple pectin and β-glucan, for which antagonism towards *S*. Choleraesuis was weaker than against *L. monocytogenes*.

## 4. Discussion

Despite glucose being commonly used as the carbon source for the cultivation of Lactic Acid Bacteria (LAB), their ability to degrade various carbohydrates is well-established and known to be strain-dependent, which was also observed in our studies [22]. Based on the results, it was concluded that the analyzed strains were able to utilize all the tested prebiotics, though they varied in relation to the type and concentration of the carbohydrates. Contrary to data presented in this study, Watson et al. (2012) showed that the growth of *Lactobacillus* spp. strains in the presence of maltodextrin and inulin was poor compared to the effect caused by glucose [23]. A similar dependency was observed by Iraporda et al. (2019) in terms of the inulin effect [24]. Also, McLaughlin et al. (2015) noted that maltodextrin, β-glucan, or corn fiber used as the carbon source for the cultivation of *Lactobacillus* spp. strains resulted in no growth or weaker growth than when glucose was used as the carbohydrate, which was opposite to our data [25]. Moreover, the researchers observed that inulin supported the growth ability of one of the ten tested *Lactobacillus* spp. strains to a comparable extent as glucose, which also indicates the strain-dependent character of these bacteria’s abilities to utilize carbohydrates [25]. Similar results were observed by Nazzaro et al. (2012), whose data showed no significant differences in the growth performance of *Lb. acidophilus* DSM 20079 when inulin, pectin, or glucose was used as the carbohydrates for the bacteria cultivation [12]. On the contrary, the results described by Kunová et al. (2011) indicate that inulin stimulated the growth of *Lactobacillus* spp. to the greatest extent among five other prebiotics [26]. The data was in line with ours for *Lb. rhamno*sus ŁOCK 1087, *Lb. reuteri* ŁOCK 1092, and *Lb. plantarum* ŁOCK 0860 cultivated in the presence of 2% (*w/v*) inulin. Furthermore, Önal Darilmaz et al. (2019) showed increased growth of tested *Lactobacillus* spp. strains in the presence of 5% (*w*/*v*) inulin compared to cultures conducted in unmodified MRS medium with 2% (*w*/*v*) glucose [27]. The results described in this article show that the selected *Lactobacillus* spp. strains have a greater ability to adapt and to degrade a wider spectrum of carbohydrates than the ones analyzed by previously mentioned researchers. Nevertheless, for the purpose of a synbiotic elaboration purposes, the inulin in the concentration of 2% (*w*/*v*) was the most beneficial for the selected strains.

Based on the metabolic profile, LAB are divided into two categories, namely homo- and heterofermentative bacteria, which degrade carbohydrates mainly into lactic acid (around 85% of the end products) or acetic acid and ethanol along with lactic acid (no less than 50% of all metabolites), respectively [28]. The results described in this article indicate the heterofermentative character of these strains, which produced lactic, acetic, and propionic acids in significantly higher concentrations than butyrate, acetaldehyde, and ethanol. According to data presented, only inulin proved to enhance the synthesis of lactate and SCFAs, which could prevent the colonization of the GIT by pathogens and improve the host’s overall health [29,30]. To this day, not many studies have been published, considering the impact of the prebiotics analyzed in this article—apart from inulin—on the metabolites produced by *Lactobacillus* spp. Nevertheless, contrary to the data obtained, Sharma and Kanwar (2018) observed a significantly lower concentration of lactic acid produced in the process of inulin fermentation than when glucose was used [31]. However, the researchers noted that inulin promoted the production of acetic, propionic, and butyric acids, which was in line with our results and the data described by de Souza Oliveira et al. (2012) [31,32]. Moreover, inulin reduced the amount of ethanol and acetaldehyde produced, which was considered beneficial since the acetaldehyde could be produced from ethanol in the process of oxidation and itself poses carcinogenic potential and could cause chromosomal mutations [33]. These results support the idea of developing a synbiotic combining the selected probiotic strains and 2% (*w*/*v*) inulin, since the main mechanism of prebiotics’ beneficial impact on health is through the enhanced synthesis of SCFAs.

Typically, the racemic mixture of both lactic acid isomers-D(−) and L(+)- is created as a result of carbohydrate fermentation; however, the ratio of the optical forms can differ depending on the carbon source and the LAB strain [34,35], which was in line with the results of this study. The accumulation of D-lactic acid can be harmful to the host, since it could lead to a D-lactic acidosis resulting in confusion, behavioral changes, ataxia, or even coma [34,36]. Moreover, D-lactate isomer could diminish the availability of L-lactic acid for neurons, for which it poses as the energy source [37], which is why the enhancement of L-lactic acid synthesis at the expense of D-lactic acid concentration caused by inulin can be considered beneficial. The data presented in this article are similar to the results obtained by Goderska et al. (2008) [38], though these researchers observed lower proportions of L-lactic acid.

Prebiotic components degradation end products, especially lactic acid and SCFAs, are known to promote the antagonistic activity of probiotic strains [39,40]. We observed that inulin used as the carbohydrate in the probiotics culture was associated with the strongest enhancement in organic acids production, as well as with the greatest antimicrobial properties, which was also concluded by Likotrafiti et al. (2015) [41]. Kanjan and Hongpattarakere (2017) observed a total reduction of viable *S*. Typhimurium SA2093 cells in the presence of both inulin and the probiotic *Lb. paracasei* I321, which agrees with our data [39]. In addition to that, Chaiyasut et al. (2017) also observed a significant reduction in *Salmonella* spp. count in the presence of inulin-degrading *Lactobacillus* spp. and the prebiotic compound [42]. Moreover, the results show that inulin also significantly stimulates the antagonistic activity of the probiotic mixture towards *L. monocytogenes,* which was the opposite of what was described by Likotrafiti et al. (2015) in their study [41]. Our results indicate that the application of the combination of the selected strains with inulin could improve the antagonistic activity towards pathogenic bacteria, inhibiting their growth and therefore preventing infectious diseases.

Furthermore, it was noted that the carbohydrates analyzed in this study did not significantly impact the enzymatic profile of the probiotic *Lactobacillus* spp. strains, although changes were noted. Independently of the carbon source, common activities of enzymes were observed for *Lactobacillus* spp., such as β-galactosidase being responsible for lactose hydrolysis, β-glucosidase impacting the ability of *Lactobacillus* spp. to endure higher temperatures and breaking glycosidic bonds, acid phosphatase helping to obtain energy from phosphates, or naphthol-AS-BI-phosphohydrolase being responsible for alkaloids, nucleotides, and proteins dephosphorylation [43,44,45,46,47]. In addition to that, all of the strains lack the activity of potentially harmful enzymes, like β-glucuronidase—which is associated with toxic, mutagenic, and carcinogenic potential—as well as chymotrypsin, which is associated with GIT diseases [47,48]. Similar enzymatic profiles of *Lactobacillus* spp. strains have been described by Pisano et al. (2014), O’Donnell et al. (2015), and Aziz et al. (2019) [48,49,50]. On the other hand, it was noted that n-acetyl-β-glucosaminidase, alkaline phosphatase, and cystine arylamidase activity were carbohydrate-dependent. Maltodextrin, corn starch, apple pectin, and β-glucan promoted n-acetyl-β-glucosaminidase activity in *Lactobacillus* spp. strains, which indicates an unfavorable change in metabolic profile, because the enzyme could cause intestinal diseases [48]. These prebiotics used as the carbon source also resulted in a lack of alkaline phosphatase activity, which constitutes an adverse effect in terms of probiotic properties of the strains, since this enzyme could improve immunomodulation and inhibit inflammatory responses in the GIT [51]. We observed that the enzymatic profiles of the selected *Lactobacillus* spp. strains only remained unchanged in the presence of inulin, compared to that exhibited by these strains when glucose was fermented. The others of the analyzed prebiotic carbohydrates induced potentially negative enzymatic activity, which could detrimentally affect health.

In summary, the replacement of glucose with inulin resulted in beneficial metabolic changes and enhanced some of the probiotic features of the *Lactobacillus* spp. strains, which was opposite to the effect caused by the rest of the analyzed prebiotics.

## 5. Conclusions

Prebiotics are thought to be selectively utilized by beneficial microorganisms and to confer a positive effect on host health. Using a combination of pro- and prebiotics, in the form of a synbiotic, should result in a more favorable effect on the host in comparison to using these components individually. Furthermore, the growth and activity of the probiotic strains should be enhanced by the prebiotic component.

Based on the results obtained, we concluded that only inulin is suitable for use as a prebiotic for the selected *Lactobacillus* spp. strains, since not only was their growth promoted, but the metabolism was also more beneficial and the antagonistic activity against pathogens intensified. Moreover, the usage of inulin as a carbohydrate in the culture medium did not have a negative impact on the enzymatic profile, a finding which was observed in the presence of the rest of prebiotics. As a result of that, a synbiotic combination of 2% (*w*/*v*) inulin with the probiotic strains could help prevent gastrointestinal infections and maintain proper pH levels in the GIT, leading to balanced microbiota. The positive changes in the amount of lactic acid and SCFAs produced as a result of inulin fermentation may have a beneficial impact on the host’s GIT health and functionality, though further in vivo studies are necessary.

## Figures and Tables

**Table 1:** Probiotic strains.

Strains	Source
*Lb. paracasei* ŁOCK 1091	Caecal content of sow
*Lb. pentosus* ŁOCK 0860	Broiler chicken dung
*Lb. plantarum* ŁOCK 0860	Plant silage
*Lb. reuteri* ŁOCK 1092	Piglet’s caecal content
*Lb. rhamnosus* ŁOCK 1087	Turkey dung

**Table 2:** The effect of the carbon source on the growth of *Lactobacillus* spp. strains.

Carbon Source ^1^	Concentration [% (*w*/*v*)]	*Lactobacillus* spp. Strain [log CFU/mL] ^2^				
		*rhamnosus* ŁOCK 1087	*paracasei* ŁOCK 1091	*reuteri* ŁOCK 1092	*plantarum* ŁOCK 0860	*pentosus* ŁOCK 1094
GLU	1.0	8.00 ± 1.19 ^a,b,c^	7.71 ± 1.03 ^a,b^	7.53 ± 1.28 ^a^	7.99 ± 1.06 ^a,b,c^	8.55 ± 1.14
	1.5	9.17 ± 0.73	8.59 ± 0.78	8.19 ± 0.72	8.52 ± 0.56	8.44 ± 0.77
	2.0	10.06 ± 0.68	10.50 ± 0.50	8.53 ± 0.84	9.70 ± 1.09	9.98 ± 0.71
MD	1.0	9.44 ± 0.93	9.89 ±0.64	8.93 ± 1.34	9.74 ± 0.98	8.79 ± 0.70
	1.5	9.70 ± 0.75	9.91 ± 1.03	9.49 ± 0.89	10.54 ± 0.50 ^b,c,d^	9.83 ± 0.46
	2.0	9.80 ± 0.97	10.37 ± 0.54	10.05 ± 0.56	10.38 ± 0.89	9.64 ± 1.15
INU	1.0	9.02 ± 0.84	9.43 ± 0.60	10.37 ± 0.52	10.15 ± 0.82	9.70 ± 1.04
	1.5	9.20 ± 1.37	9.61 ± 0.92	11.04 ± 0.37 ^d^	10.48 ± 0.80	10.08 ± 1.06
	2.0	10.91 ± 0.92 ^c,d^	10.46 ± 0.59	11.09 ± 0.47 ^d^	10.71 ± 0.81 ^c,d^	10.38 ± 1.14
CS	1.0	8.74 ± 0.30	8.24 ± 0.78	8.24 ± 0.63	9.58 ± 0.80	8.42 ± 0.67
	1.5	9.60 ± 0.89	8.99 ± 0.46	9.55 ± 0.80	10.18 ± 0.59	9.68 ± 1.14
	2.0	10.10 ± 0.72	9.00 ± 1.01	9.26 ± 1.18	10.38 ± 0.69	9.47 ± 1.00
AP	1.0	8.92 ± 1.04	9.57 ± 0.87	10.01 ± 0.29	10.14 ± 0.72	10.12 ± 1.06
	1.5	9.43 ± 0.71	9.70 ± 0.75	10.51 ± 0.57	10.55 ± 0.76 ^b,c,d^	10.84 ± 0.15 ^c.d^
	2.0	9.86 ± 0.85	9.93 ± 1.01	9.93 ± 1.01	10.12 ± 1.10	9.90 ± 1.20
BG	1.0	8.33 ± 1.05	9.43 ± 0.62	8.64 ± 0.84	8.44 ± 0.80	8.97 ± 0.67
	1.5	9.23 ± 0.67	9.41 ± 0.75	9.24 ± 1.29	9.02 ± 0.74	8.85 ± 1.15
	2.0	9.21 ± 0.97	10.33 ± 0.46	9.88 ± 0.32	10.12 ± 0.57	9.43 ± 0.80

^1^ Carbohydrates used for the strains culture: GLU–glucose, MD–maltodextrin, INU–inulin, CS-corn starch, AP–apple pectin, and BG–β-glucan. ^2^ Lowercase letters (a–d) represent significantly different mean values, which were determined by multi-way ANOVA (*p* < 0.05) followed by post hoc Tukey’s test. The analysis included the impact of all categorical variables, namely, the probiotic strain, the type of prebiotic, and its concentration, as well as their interaction on the results. For clear data presentation, values that were similar to other results showed in the table, and should be categorized as “a,b,c,d” were left unlabeled.

**Table 3:** Influence of prebiotics on the synthesis of optically active forms of lactic acid by *Lactobacillus* spp. strains.

*Lactobacillus* spp. Strain	Lactic Acid Isomer	Carbon Source ^1^					
		GLU	MD	INU	CS	AP	BG
		Concentration [g/l] ^2^					
*rhamnosus* ŁOCK 1087	D(-)	0.942 ± 0.027 ^d, e^	0.119 ± 0.008 ^a, b^	0.402 ± 0.075 ^a, b, c, d^	0.087 ± 0.004 ^a^	0.166 ± 0.048 ^a, b^	0.088 ± 0.014 ^a^
	L(+)	4.570 ± 1.106 ^g^	0.407 ± 0.003 ^a, b, c^	6.634 ± 0.996 ^h, i^	0.106 ± 0.003 ^a, b^	0.083 ± 0.007 ^a^	0.105 ± 0.009 ^a, b^
	Total	5.512 ± 1.079 ^B^	0.527 ± 0.011 ^A^	6.634 ± 0.921 ^B^	0.193 ± 0.001 ^A^	0.249 ± 0.019 ^A^	0.193 ± 0.022 ^A^
*paracasei* ŁOCK 1091	D(-)	4.511 ± 0.412 ^g^	0.112 ± 0.015 ^a, b^	2.200 ± 0.301 ^f^	0.023 ± 0.002 ^a^	0.094 ± 0.003 ^a^	0.049 ± 0.006 ^a^
	L(+)	2.424 ± 0.110 ^f^	0.547 ± 0.019 ^a, b, c, d, e^	5.701 ± 0.105 ^h^	0.032 ± 0.002 ^a^	0.169 ± 0.049 ^a, b^	0.057 ± 0.005 ^a^
	Total	6.935 ± 0.522 ^C^	0.659 ± 0.004 ^B^	7.901 ± 0.196 ^D^	0.056 ± 0.005 ^A^	0.263 ± 0.046 ^A, B^	0.106 ± 0.012 ^A^
reuteri ŁOCK 1092	D(-)	0.950 ± 0.029 ^e^	0.091 ± 0.007 ^a^	0.517 ± 0.111 ^a, b, c, d, e^	0.025 ± 0.002 ^a^	0.026 ± 0.001 ^a^	0.017 ± 0.001 ^a^
	L(+)	4.698 ± 0.381 ^g^	0.472 ± 0.001 ^a, b, c, d, e^	7.282 ± 0.206 ^j^	0.076 ± 0.009 ^a^	0.071 ± 0.009 ^a^	0.078 ± 0.005 ^a^
	Total	5.648 ± 0.411 ^C^	0.563 ± 0.007 ^B^	7.799 ± 0.317 ^D^	0.101 ± 0.011 ^A^	0.097 ± 0.009 ^A^	0.095 ± 0.005 ^A^
*plantarum* ŁOCK 0860	D(-)	1.003 ± 0.014 ^e^	0.067 ± 0.002 ^a^	0.501 ± 0.007 ^a, b, c, d, e^	0.008 ± 0.001 ^a^	0.034 ± 0.005 ^a^	0.020 ± 0.003 ^a^
	L(+)	6.285 ± 0.089 ^i^	0.487 ± 0.013 ^a, b, c, d, e^	7.044 ± 0.089 ^j^	0.059 ± 0.002 ^a^	0.095 ± 0.002 ^a^	0.085 ± 0.011 ^a^
	Total	7.287 ± 0.076 ^C^	0.554 ± 0.011 ^B^	7.546 ± 0.082 ^D^	0.068 ± 0.009 ^A^	0.129 ± 0.017 ^A^	0.104 ± 0.008 ^A^
*pentosus* ŁOCK 1094	D(-)	0.798 ± 0.085 ^c, d, e^	0.051 ± 0.040 ^a^	0.284 ± 0.052 ^a, b, c^	0.046 ± 0.005 ^a^	0.085 ± 0.004 ^a^	0.027 ± 0.002 ^a^
	L(+)	4.237 ± 0.183 ^g^	0.642 ± 0.084 ^b, c, d, e^	7.248 ± 0.304 ^j^	0.074 ± 0.011 ^a^	0.061 ± 0.005 ^a^	0.054 ± 0.004 ^a^
	Total	5.035 ± 0.103 ^C^	0.692 ± 0.44 ^B^	7.531 ± 0.252 ^D^	0.120 ± 0.016 ^A^	0.146 ± 0.001 ^A^	0.081 ± 0.008 ^A^

^1^ Carbohydrates used for the strains culture: GLU–glucose, MD–maltodextrin, INU–inulin, CS-corn starch, AP–apple pectin, and BG–β-glucan. ^2^ Lowercase letters (a–j) were used to mark significantly different concentrations of lactic acid isomers, which was determined by multi-way Welch-ANOVA (*p* < 0.05) with the post hoc Tukey’s test. Three different categorical variables were included, namely, type of the carbohydrate, strain, and isomer. For each strain separately, significantly different total lactic acid concentration mean values were labeled with different uppercase letters (A–D) following performed one-way Welch–ANOVA (*p* < 0.05) with post hoc Tukey’s test.
